# The cancer cell secretome drives cooperative manipulation of the tumour microenvironment to accelerate tumourigenesis

**DOI:** 10.12703/r/10-4

**Published:** 2021-01-19

**Authors:** Shona Ritchie, Daniel A Reed, Brooke A Pereira, Paul Timpson

**Affiliations:** 1 The Kinghorn Cancer Centre, Garvan Institute of Medical Research, Sydney, New South Wales 2010, Australia; 2 St. Vincent's Clinical School, Faculty of Medicine, University of New South Wales, Sydney, New South Wales 2010, Australia

**Keywords:** Cancer cell secretome, Tumour microenvironment, Stroma, Pre-metastatic niche

## Abstract

Cellular secretions are a fundamental aspect of cell–cell and cell–matrix interactions *in vivo*. In malignancy, cancer cells have an aberrant secretome compared to their non-malignant counterparts, termed the “cancer cell secretome”. The cancer cell secretome can influence every stage of the tumourigenic cascade. At the primary site, cancer cells can secrete a multitude of factors that facilitate invasion into surrounding tissue, allowing interaction with the local tumour microenvironment (TME), driving tumour development and progression. In more advanced disease, the cancer cell secretome can be involved in extravasation and metastasis, including metastatic organotropism, pre-metastatic niche (PMN) preparation, and metastatic outgrowth. In this review, we will explore the latest advances in the field of cancer cell secretions, including its dynamic and complex role in activating the TME and potentiating invasion and metastasis, with comments on how these secretions may also promote therapy resistance.

## The cancer cell secretome

The term “secretome” can be defined as any factor (soluble or insoluble) that is released or secreted into the extracellular space^1–4^. The secretome can consist of a diverse array of factors including chemokines, cytokines, growth factors, coagulation factors, hormones, enzymes, glycoproteins, and microRNAs (miRNAs). These factors can be secreted individually or contained in vesicles such as extracellular vesicles (EVs) (e.g. exosomes) or nanovesicles (e.g. exomeres)^5–8^. In normal tissues, the secretome is tightly regulated to maintain tissue homeostasis, with secreted proteins and their cognate receptors functioning as the main mechanism by which cells and tissues communicate^4,9^. The cell secretome is diverse in its function, where secreted proteins can act in an autocrine, paracrine, or endocrine manner, both locally and systemically. Soluble proteins are synthesised as precursors at the endoplasmic reticulum (ER) and then transported to the Golgi apparatus, where they are packaged and excreted^10,11^. This “classical” secretory process is completed when secretory vesicles fuse with the plasma membrane and their contents are expelled from the cell surface into the microenvironment^10^. Several other “non-classical” pathways of secretion have also been described, where synthesised proteins may be secreted directly from the ER in a Golgi-independent manner^11^. Exosomes, for example, are composed of intraluminal vesicles inside multivesicular bodies which fuse with the plasma membrane independent of the Golgi^11,12^.

**Figure 1.**
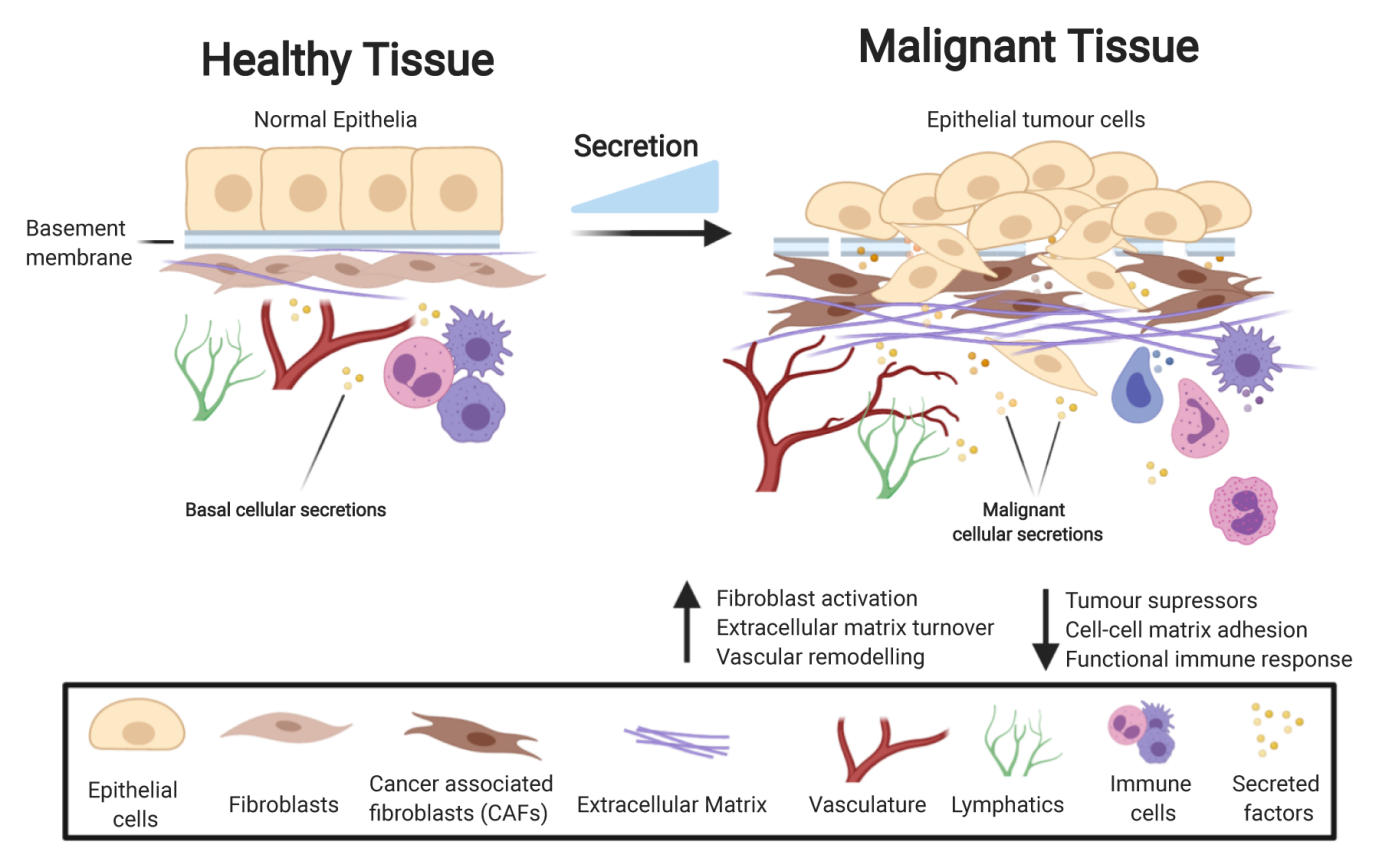
The cancer cell secretome drives a pro-tumourigenic environment. During the process of cancer development, the secretome is markedly changed compared to healthy tissue, with increased levels of secretion resulting in a change to many key processes enhancing tumour growth. Examples of pathways affected by tumour cell-derived secretion include an increase in fibroblast activation, extracellular matrix (ECM) deposition, and vascular remodelling while key pathways in tumour suppression, cell–cell matrix adhesion, and regulation of the immune system are lost.

Recent revision of the human secretome^4^ has found that approximately 13% of all human protein-coding genes code for secreted proteins. However, for some secretory organs such as the pancreas and salivary gland, secreted proteins account for the majority of transcriptional outputs, as expected^13^. In cancer, the secretome is significantly altered^14–16^, often displaying widespread changes across multiple cellular processes. A recent pan-cancer analysis conducted by Robinson *et al*. established that the cancer cell secretome is markedly distinct from paired healthy tissues^17^. In this analysis, secretome transcriptomic signatures of 32 cancer types were compared with 30 healthy counterparts, specifically comparing genes that were expected to be most differentially expressed between different cancer types and healthy participants. Interestingly, the subset of the secretome that exhibited the strongest differential expression across the majority of the cancer types included loss of tumour suppressors (putative or established) as well as loss of genes involved in cell–cell/matrix adhesions and the immune response^17^. Strikingly, when assessing for transcripts which were overexpressed across the cancer cohort, most top-ranking genes were related to extracellular matrix (ECM) structure, composition, and modification as well as vascular remodelling, although there were less shared transcript commonalities between different cancers for this analysis^17^. Overall, this systematic study highlights the dynamic, complex nature of the cancer cell secretome and its role in the pathophysiology of cancer (Figure 1). It suggests that there is a common global shift towards pro-tumourigenic pathways, including ECM and vascular remodelling and loss of tumour suppressors; however, it is important not to overlook other secretome pathways in specific cancers. Large-scale studies such as these are interesting to gain a global perspective, but care should be taken not to over-speculate, so as not to miss critical cancer type-specific nuanced factors that can drive progression.

## The cancer cell secretome activates the tumour microenvironment

### Basement membrane degradation

**Figure 2.**
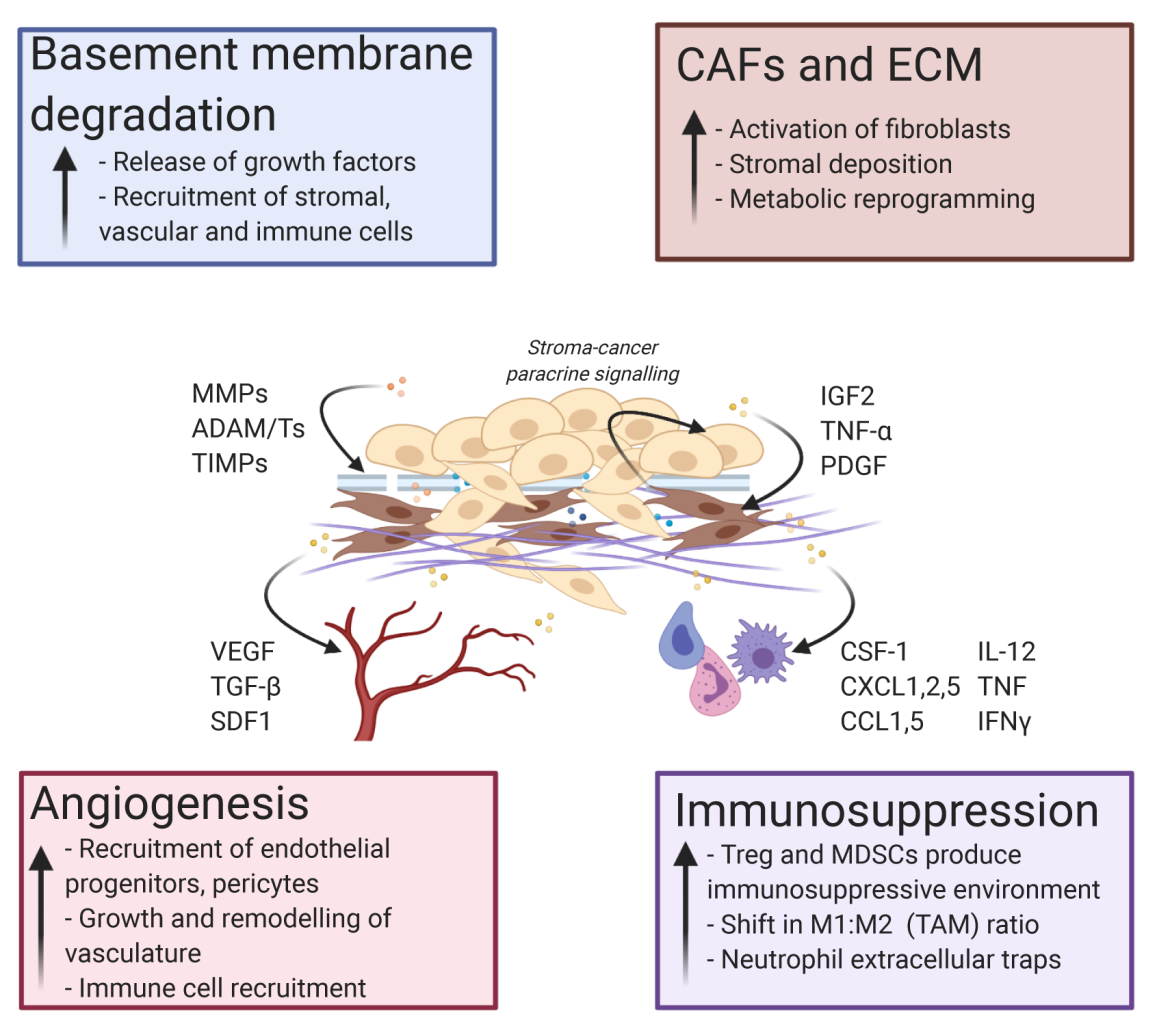
The cancer cell secretome drives tumour microenvironmental changes enhancing tumour growth, invasion, and metastasis. Tumour cell secretion activates a number of pro-tumourigenic processes. These events include basement membrane degradation, activation of cancer-associated fibroblasts (CAFs), deposition of extracellular matrix (ECM), angiogenesis at the tumour site, and immunomodulation to favour an immunosuppressive environment. Examples of key tumour-secreted proteins that affect each major process are shown. ADAM/T, a disintegrin and metalloproteinase/with thrombospondin motif; CCL, C-C motif chemokine ligand; CSF-1, colony-stimulating factor 1; CXCL, C-X-C motif chemokine ligand; IFN, interferon; IGF2, insulin-like growth factor 2; IL, interleukin; MDSC, myeloid-derived suppressor cell; MMP, matrix metalloproteinase; PDGF, platelet-derived growth factor; SDF1, stromal cell-derived factor 1; TAM, tumour-associated macrophage; TGF, transforming growth factor; TIMP, tissue inhibitor of metalloproteinase; TNF, tumour necrosis factor; Treg, regulatory T cell; VEGF, vascular endothelial growth factor.

During early malignancy, reciprocal heterotypic paracrine signalling between tumour cells and other tumour microenvironment (TME) components triggers a cascade of biochemical and biomechanical changes, creating a dynamic niche of cell–cell and cell–matrix interactions. For many solid cancers, a prerequisite for the initiation of malignancy involves the secretion of ECM remodelling enzymes by newly transformed tumour cells to degrade the basement membrane (BM) (Figure 2)^18^. The metalloproteinase families are the main enzymes within the secretome that carry out ECM degradation^18,19^. Two branches exist: matrix metalloproteinases (MMPs) and a disintegrin and metalloproteinase/with thrombospondin motifs (ADAM/Ts), both of which are frequently found to be overactivated in cancer^20–22^. MMPs are zinc-binding endopeptidases with varying targets within the ECM^23^. For example, MMP1 digests collagen III, MMP3 and MMP10 prefer fibronectin and laminins, and MMP2 and MMP9 break down gelatine^23^.

The role of MMPs in cancer are considered highly context dependent, exhibiting both tumour-promoting and -restraining roles^24,25^. For instance, MMPs and ADAMs can release cell membrane precursors for growth factors such as insulin-like growth factor (IGF) and epidermal growth factor receptor (EGFR) ligands, resulting in enhanced proliferation^26^. Early work on broad-range MMP inhibition in cancer was challenging and did not progress into clinical trials owing to insufficient selectivity, often impacting MMPs involved in other physiological processes and other zinc-dependent proteases^27^. Much effort is now concentrating on designing next-generation agents capable of discriminating between endogenous and disease-inducing MMPs^28^. For example, in pre-clinical models of pancreatic cancer, it was shown that blocking Src activity by dasatinib treatment reduced the activity of MMP2 and MMP9 and slowed metastasis^29^. MMP enzymatic activity can also be regulated by endogenous proteins within the TME, known as tissue inhibitors of metalloproteinases (TIMPs), which are often found to be lost in several cancers^30^. Interestingly, Scilabra *et al*. recently found that TIMP3 overexpression decreases shedding of ADAM10 substrates^31^. The metalloproteinase ADAM10 has been reported to shed several cancer-promoting proteins, from which downstream signalling can activate pathways such as Notch and Eph, which have been shown to induce tumour growth and chemoresistance^32^. Therefore, reduction of ADAM10-shed substrates by TIMP3 overexpression was expected to be beneficial. However, Scilabra and colleagues showed this interaction simultaneously increased the expression of several other secreted proteins such as SPARC^31^, a well-characterised ECM protein implicated in several cancers^33,34^. This evidence therefore suggests that careful consideration of proteolytic therapy in cancer must be taken, exemplifying how it can dramatically and unexpectedly alter secretome behaviour and ECM composition^31^. Overall, and without targeted inhibition, MMPs, ADAMs, and TIMPs work collectively to cleave and degrade ECM molecules, which allows for invasion of tumour cells beyond the BM. MMP family members have also been shown to participate in other cancer-promoting actions like mediating communication between the tumour and stroma^35^, allowing for paracrine signalling to recruit and activate fibroblasts, vascular cells, and immune cells. Activated stromal cells then signal back to the tumour cells and the TME, resulting in a complex, pro-tumourigenic loop (Figure 2).

### The cancer secretome influences angiogenesis

In cancer development, newly formed tumours will initially utilise pre-existing vasculature to proliferate and expand^36–38^. However, soon cancer cells will begin to recruit endothelial progenitors and supporting pericytes to the TME *via* secreted factors to grow and remodel new vasculature, termed angiogenesis (Figure 2)^39–42^. Cancer cell hypoxia has become a well-established phenomenon for inducing angiogenesis, where hypoxia-driven pH changes to the TME can result in the recruitment of vascular cells by cancer cell-secreted proteins such as vascular endothelial growth factor (VEGF), transforming growth factor-beta (TGF-β), stromal cell-derived factor 1 (SDF-1/CXCL12), and angiopoietins as well as genetic material such as miRNAs (Figure 2)^42–45^. Mounting evidence is showing that many of these factors are secreted from cancer cells in EVs and that the acidic TME might encourage the production, release, and survival of EVs to encourage angiogenic growth^46^. Particularly, several EV-packaged miRNAs have been described in different cancers, where they are received by endothelial cells to promote the proliferation of new vessel growth and therefore migration. Hsu and colleagues described this process in hypoxic lung cancer cells, where they showed increased exosomal secretion in hypoxic compared to normoxic conditions^47^. Within the exosomes, upregulated miR-23 expression targeted hypoxia-inducible factor 1-alpha (HIF1α) to promote pro-angiogenic activities of endothelial cells^47^. In glioblastoma, hypoxic cancer cells upregulated the secretion of miR-182-5p, stimulating a potent accumulation of VEGF receptor (VEGFR) and repression of tight junction molecules. This combination resulted in enhanced angiogenesis and increased permeability of further exosomes^48^. Tumour cell-derived exosomes may also target other stromal cells to encourage the secretion of pro-angiogenic factors. Breast cancer exosomes targeted adipocyte-derived mesenchymal stem cells to transform them into a myofibroblast-like phenotype, resulting in the increased secretion of VEGF, SDF-1, and TGF-β^49^. This cancer cell-derived exosomal paracrine pathway resulted in the upregulation of angiogenic pathways, with myofibroblasts acting as the intermediate player^49^. Strikingly, Follain and colleagues have highlighted that extravasation and endothelial remodelling is partly blood flow dependent and leads to increased metastases^50,51^. Their studies revealed that the vascular endothelium wall was actively remodelled around the extravasating circulating tumour cell (CTC), and hemodynamic cues from the sheer force of blood flow activated VEGFR pathways to encourage the exit of the cancer cell towards a metastatic site^51^. Thus, although this review focusses on the cancer secretome, the work by Follain *et al*. is one of many studies that showcases the importance of other dynamic physiological cues that also contribute towards developing a cancer-permissive environment^8^. As well as this, angiogenesis allows increasing numbers of immune cells to infiltrate and modulate the immunosurveillance landscape through increased permeability^52,53^ (Figure 2). This reiterates the complex cancer paracrine pathways that utilise the cancer cell-activated microenvironment. Overall, secretions from cancer cells dictate the initiation and maintenance of pro-angiogenic pathways that allow for tumour growth during altered physiological conditions. These new vessels also provide an increased likelihood for primary tumour cells to migrate towards the systemic vasculature, where they may begin their journey towards a secondary site.

### Cancer cell secretome and immunomodulation

The cancer cell secretome is critical to promoting immunosuppression in the TME. Immunosuppression can occur when cytotoxic T cells are impeded by other immune cell populations, such as tumour-associated macrophages (TAMs), regulatory T cells (Tregs), and myeloid-derived suppressor cells (MDSCs)^54^, or are exhausted because of prolonged cancer cell antigen presentation (Figure 2)^55^. The roles of immune cells can also be dictated by cancer cells throughout tumour progression. TAMs, for example, are in a state of potential flux and can be primed to switch between an M1/M2 state by the cancer secretome. In simplified terms, TAMs in a pro-inflammatory M1-like state can be recruited to the tumour site by cytokines such as interleukin 12 (IL-12), tumour necrosis factor (TNF), and interferon gamma (IFNγ) during early oncogenesis^56^. Initially, TAMs in this state are believed to be anti-tumourigenic, releasing cytotoxic agents that damage cancer cells such as nitric oxide (NO), and can also destroy malignant cells by engulfing them^56,57^. However, prolonged TAM activity can eventually cause chronic inflammation and genomic instability in neoplastic cells, promoting malignant proliferation and progression. Additionally, cancer cells can repolarise TAMs towards an M2 state through secretion of metabolism re-programming factors, such as colony-stimulating factor 1 (CSF-1), and metabolites, such as lactate^56^. In these conditions, TAMs are then able to secrete pro-tumourigenic factors that can further modulate the TME such as VEGF (pro-angiogenic), IL-10 (immunosuppressive), EGF (growth promoting), and MMPs (matrix remodelling)^58^ (Figure 2). Importantly, a complex mosaic of TAMs in M1/M2 states can occur spatially and temporally in any given tumour, which therefore promotes different conditions of inflammation and immune surveillance within the same tumour area. Further interrogation of fluctuating TAM polarisation should therefore be considered for future drug targets. Neutrophils can also be activated by the cancer cell-derived secretions, with a recent study by Teijeira *et al*. reporting that cancer cell-derived chemokines (IL-8, CXCL1, CXCL2, CXCL8) activate the CXCR1 and CXCR2 receptors on neutrophils, resulting in the formation of neutrophil extracellular traps (NETs)^59^. NETs act to physically shield cancer cells from the immune system, in particular cytotoxic T cells and NK cells, promoting cancer growth^59^. NETs have also been implicated in stimulating dormant cancer cells^60^, pre-metastatic niche (PMN) formation^61^, and alteration of mitochondrial activity^62,63^. Furthermore, inhibition of neutrophil-upregulated CXCR2 expression improved response to checkpoint inhibitors, which slowed tumourigenesis, suppressed metastasis, and improved infiltration of cytotoxic T cells in pancreatic cancer^64^. This suggests that neutrophils have diverse tumour-promoting functions and have therapeutic targeting potential.

MDSCs also play a significant role in promoting immunosuppression in the TME. MDSCs are recruited from the bone marrow to the tumour site *via* cancer cell-derived chemokines such as CCL2, CCL5, CXCL5, and IL-8^54^. Here, they initiate several immunosuppressive processes, which impact other immune cells in their surroundings, such as nutrient deprivation of T cells^65^. MDSCs can also cause inhibition of lymphocyte homing, where the production of damaging molecules such as reactive oxygen species (ROS) and NO inhibit the expression of immune checkpoint molecules^66^. As well as during cancer progression, MDSCs have been shown to utilise these tactics to promote cancer relapse during chemotherapy^67^. Rong and colleagues showed that doxorubicin (Dox)-resistant breast cancer cells secrete prostaglandin E2 (PGE2) to support the expansion of MDSCs^67^. This results in inhibition of CD4^+^CD25^–^ T cells and enhanced immune chemotherapy resistance^67^. MDSCs also interact with cells of the adaptive immune system such as Tregs to impede immunosurveillance. One study has shown that MDSCs must first be activated in the TME to permit the differentiation and infiltration of Tregs^68^. However, more recently, Lee *et al*. provided evidence that Tregs can modulate MDSC expansion and function through TGF-β^69^. Tregs suppress the inflammatory response and control anti-cancer immunity and are identified by the expression of the master transcription factor forkhead box protein p3 (FOXP3). They remain a difficult subset of T cells to target owing to commonality with cytotoxic T cells, which are generally protective^70^. As well as paracrine recruitment *via* other immune cells, cancer cells can directly recruit Tregs to initiate anti-cancer immunity. For example, in pancreatic ductal adenocarcinoma (PDAC), Wang *et al*. found that FOXP3-positive cancer cells secreted CCL5 to recruit Tregs into the TME, which can be blocked to repress Treg influx and tumour growth^71^. Thus, cancer-promoting secretions activate several interactive immune pathways to ultimately shield the invading tumour cells from a functional immune response.

### Cancer cell secretome activates cancer-associated fibroblasts and alters the extracellular matrix

Cancer cell-derived secretions also recruit mesenchymal stem cells (MSCs) and fibroblasts, which become activated to form heterogenous populations of cancer-associated fibroblasts (CAFs) capable of distinct cancer-promoting functions^72–77^ (Figure 2). CAFs are the most abundant cell type in the tumour stroma and have far-reaching effects in the TME, where they can act to both restrain^78–81^ and promote tumour development and progression^72,75,82,83^. CAFs can also indirectly influence tumour progression through the regulation of metabolic reprogramming, angiogenesis, and inflammation in the TME^84–87^ (Figure 2).

Crucially, CAFs are the main producers of structural ECM components such as fibrillar collagens, fibronectin, and hyaluronic acid^88,89^. ECM is commonly dysregulated across a number of solid malignancies^90^. ECM molecules accumulate as CAFs proliferate in early cancer development and contribute to an altered ECM composition, porosity, and topography as well as increases in tissue stiffness^88,91,92^. Thus, cancer matrix remodelling consists of a juxtaposition of processes: stromal cell recruitment and proliferation leads to excessive deposition of large ECM components such as collagens and proteoglycans, while the simultaneous breakdown of ECM elements from cancer cell-derived secretory factors allows for BM degradation and the process of invasion to be initiated. ECM provides both biochemical and biomechanical cues to promote cancer^93–95^. The ECM can directly influence tumour cells but also indirectly promote angiogenesis, inflammation, and further stromal activation^73,76,93,96^. ECM is also implicated in the preparation of the metastatic niche and metastatic outgrowth, which will be discussed later in this review. Interestingly, Oudin and colleagues found that cancer-driven ECM remodelling promotes haptotaxis (gradient-directed motility) of cancer cells up a fibronectin gradient and towards the bloodstream^97^. The study found that the integrin α_5_β_1_ interacts with an isoform of an actin-regulating protein, MENA^Inv^, which allows the cells to move towards an increased concentration of fibronectin closer to the perivascular compartment^97^. Although this mechanism is tumour cell intrinsic, it manipulates the ECM in such a way that it provides a remodelled path for the cancer cells to travel through, thereby encouraging metastasis.

Aberrant ECM in malignancy is generally thought to be secreted by stromal cells such as CAFs. This was partially disputed by Tian *et al*., who assessed the contribution of ECM deposition from stromal cells and epithelial cancer cells in pancreatic ductal adenocarcinoma (PDAC) using a cross-species identification approach^98^. Here, the authors report that over 90% of tumour-associated ECM is produced by stromal cells, with the other 10% being tumour cell-derived ECM proteins^98^. The authors implied that proteins originating from CAFs were associated with both anti- and pro-tumourigenic elements, whereas cancer cell-derived ECM were associated with pro-tumourigenic actions more frequently^98^. Although this is an interesting concept that should be explored further, it is important to note that this work was performed with transplanted human PDAC tumours grown in a murine host, where they identified the source of ECM by species, thereby appointing tumour-derived ECM from humans and all stromal-derived ECM as mouse. Care should be taken to not solely attribute pro-tumourigenic ECM characteristics to only one compartment, i.e. the cancer cells, as CAFs also feedback to cancer cells to promote tumour progression and development. Overall, the reciprocal heterotypic communication between tumour and stromal cells allows continual synthesis, production, and secretion of an abundance of transformative proteins, which ultimately aids tumour invasion and metastasis. The studies discussed have underscored the complexity of tumour–TME crosstalk, and the need for a better understanding of the dynamic environment, in order to improve the efficacy of anti-stromal therapies in desmoplastic cancers.

## The cancer cell secretome promotes preparation of the pre-metastatic niche, metastatic migration, and outgrowth

The process of metastasis involves many levels of evasion by cancer cells and their associated secreted factors. Survival in the systemic vasculature is relatively unlikely for cancer cells^8,99^, and successful growth at secondary sites relies on malignant cells to dynamically shape the new “ecosystem” before and during expansion. Metastatic niches can either be established as CTCs arrive at a specific metastatic site or prepared in advance by primary or CTC-secreted factors, resulting in the development of the PMN^100,101^. The precise secretome and molecular mechanisms of the PMN vary greatly between cancer types but, in general, can be broken down into a series of steps, beginning with BM breakdown, alteration of resident cells in the target organ, remodelling of the PMN ECM, recruitment of non-resident cells such as bone marrow-derived haematopoietic progenitor cells and widespread systemic recruitment of immune cells^8,10^. Cancer cells can also educate each other to become more migratory and pro-metastatic. For example, Zomer *et al*. reported that malignant tumour cells can trigger migratory behaviour and metastatic capacity in tumour cells which were less malignant by short- and long-range exchange of EVs *in vivo*^102^. A later study by the same group established that the highly malignant EVs contain both RNA and proteins enriched for migratory behaviour^103^. This is further supported by a study by Gangoda *et al*., who reported that cells with varying metastatic potential had differential exosomal cargo, which could then facilitate metastatic outgrowth at distinct sites^104^. Similarly, Kalra *et al*. showed that EVs can transfer mutant β-catenin to the recipient cells, promoting cancer progression *via* activation of Wnt signalling^105^. Several eloquent reviews discuss the influence of the cancer secretome at each step in detail^99,101,106^. This review will highlight the most recent findings regarding the tumour secretome in organotropism, PMN preparation, and metastatic maturity.

### Organotropism in metastasis

**Figure 3.**
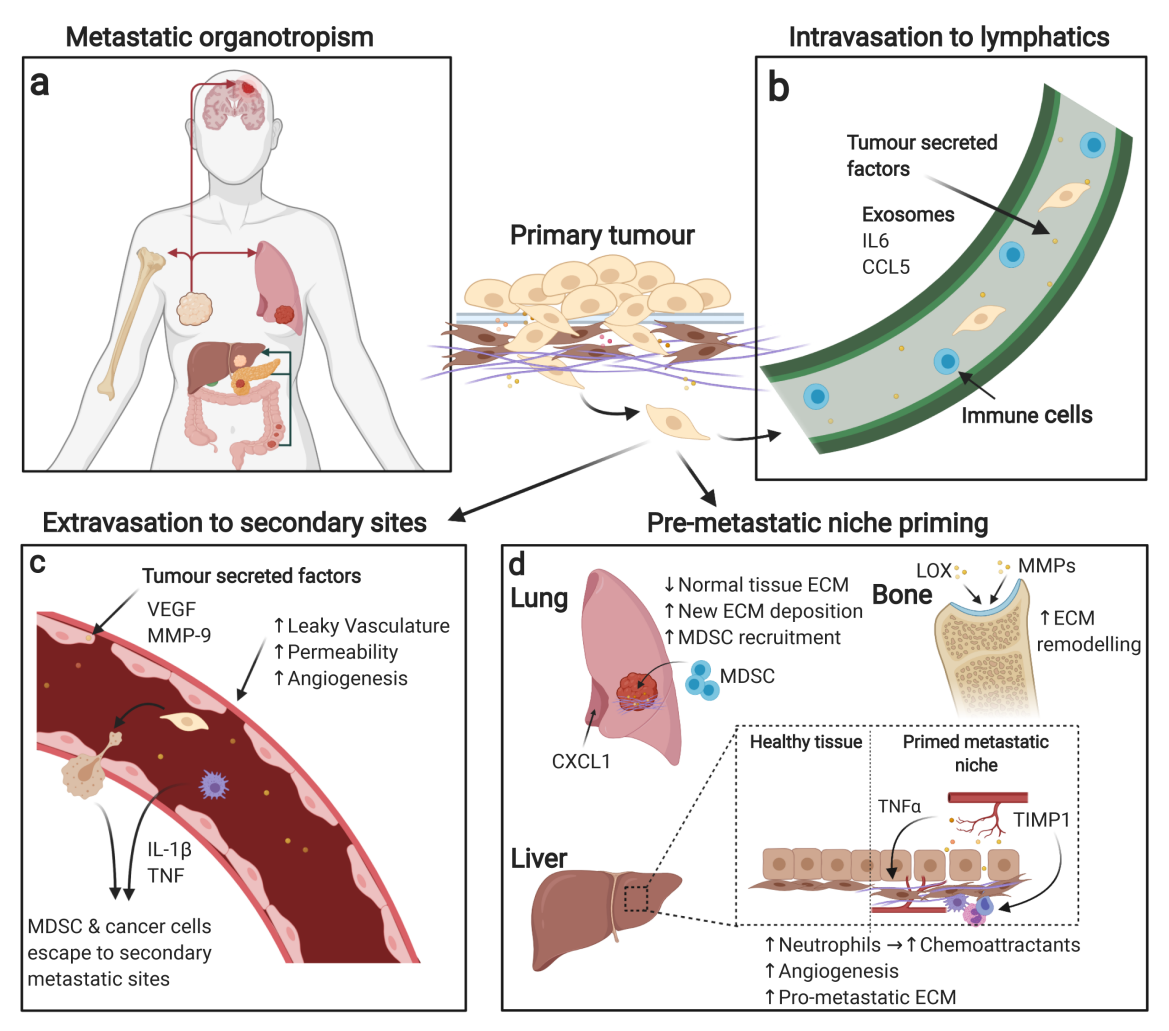
The tumour secretome activates pro-metastatic events. Successful tumour cell metastasis and survival at secondary sites is a multi-step process involving many different mechanisms. (**a**) Tumour cells originating from different primary tumour sites display organotropism for secondary metastasis sites; examples of this include breast cancer’s favourable growth in the brain, bone, and lung as well as pancreatic and colon cancer’s favourable growth in the liver. (**b**) Tumour cells initially preferentially metastasise to the lymphatic system owing to a favourable environment where they can then spread to distant secondary organs. (**c**) Tumour-derived secreted proteins can provide pro-metastatic signals during vasculature remodelling. For example, tumour-secreted proteins can induce leaky vasculature, increasing permeability for tumour cells and other cells to access secondary sites. (**d**) Metastatic niche priming can also be affected by primary tumour secretions, with examples of priming including extracellular matrix (ECM) remodelling, immune system recruitment, vascular remodelling, and chemoattractant secretion at metastatic sites. CCL5, C-C motif chemokine ligand 5; CXCL1, C-X-C motif chemokine ligand 1; IL, interleukin; LOX, lysyl oxidase; MDSC, myeloid-derived suppressor cell; MMP, matrix metalloproteinase; TIMP, tissue inhibitor of metalloproteinase; TNF, tumour necrosis factor; VEGF, vascular endothelial growth factor.

Common sites of metastasis can include the lymph node, bone, liver, lung, brain, and peritoneum^107^. Depending on the origin of the primary tumour, specific organs are more prone to PMN transformation and primary metastatic maturity (Figure 3a). Recent work by Hoshino *et al*. showed that exosomes released by metastatic cancer cells preferentially precondition resident cells at their PMN organ choice in an integrin-dependent manner^108^. Remarkably, they also showed that breast cancer cell-derived exosomes (that colonise to the lung) could redirect the disseminating cancer cells of a different type from its usual PMN choice of bone and instead metastasise to the lung^108^. They suggested this behavioural change may be down to instructive integrins expressed on the exosomes which could direct the migration of cancer cells. For example, integrin α_v_β_5_ directed cells to the liver, whereas α_6_β_4_ encouraged migration to the lung^108^. This was also shown in pancreatic cancer by Costa-Silva *et al*., who observed that pancreatic cancer exosomes can direct PMN establishment to the Kupffer cells of the liver, and is discussed more later in the review^109^. Therefore, although these concepts require further study, it showcases novel potential mechanisms for cancer cell-directed organotropism in metastasis.

Epithelial-derived cancers often initially extravasate into the lymphatic system (Figure 3b), where CTCs encounter immune cells that are programmed to modulate future immune responses, as extravasation from the primary tumour continues^110^. Lymph node metastasis predicts poor patient outcomes in several cancers including breast, prostate, lung, melanoma, and colorectal cancers, as it signifies high probability that malignancy will spread to a number of organs from lymphatic circulation^8^. Recently, Ubellacker and colleagues revealed research to explain why it is common for cancer cells, as in melanoma, to first metastasise to the lymph rather than directly into the bloodstream^111^. As previously mentioned, after cancer cells extravasate into the systemic vasculature (Figure 3c), most of them die owing to the extreme change of environment, where they battle with loss of ECM adhesion, shear stress from blood flow, and impacting red blood cells^112^. Additionally, ROS molecules are mediators of cancer cell death within the circulation. Ubellacker *et al*. showed that tumour cells circulating the systemic vasculature are killed by a form of ROS, known as ferroptosis, which is an iron-dependent death pathway that results in lipid peroxidation^111^. Therefore, to avoid this, metastatic melanoma cells were suggested to first congregate at the lymph nodes as a survival mechanism. This elegant study by Ubellacker *et al*. demonstrated that melanoma cells that first move through lymph followed by circulatory exit are more likely to survive than melanoma cells that enter the bloodstream directly^111^. At lymphatic metastases, other studies have also shown that cancer cells successfully move into the circulation *via* perfusion^113,114^. Before the arrival of CTCs in lymph nodes, however, the tissue has been primed by secretions from primary tumour cells. For example, tumour cell-secreted IL-6 from triple negative breast cancer (TNBC) cells triggers STAT3 phosphorylation in lymphatic endothelial cells^115^. This leads to a cascade of transcriptional events that results in CCL5 expression in the lymphatic vasculature, allowing for the recruitment and infiltration of tumour cells^115^.

Breast cancer, along with gastrointestinal cancers such as pancreatic and colorectal, also commonly prime the liver for PMN signal cascades (Figure 3d)^108^. The ECM remodelling enzyme TIMP1, for example, was shown to be secreted from colorectal cancer cells to induce SDF-1 upregulation at the liver, which recruits neutrophils that in turn secrete factors that act as a chemoattractant for tumour cells^116,117^. Similarly, we recently showed that cancer cells that harbour a gain-of-function *TP53* mutation have enhanced TNFα/NFκB paracrine signalling in pre-clinical models of PDAC^82^, which in turn educates adjacent CAFs, genetically tuning them to secrete aberrant levels of perlecan, which is pro-metastatic and chemoprotective at the liver^82^. Furthermore, the previously mentioned study by Costa-Silva and colleagues reported that Kupffer cells in the liver selectively uptake malignant pancreatic exosomes containing pro-fibrotic signalling molecules (TGF-β and fibronectin), supporting liver-specific PMN formation in PDAC^109^. Specifically, they showed that exosomal macrophage migration inhibitory factor (MIF) induced these changes in the liver and that this was conserved in human PDAC cases (Figure 3d)^109^.

Bone is often another common target of PMN from primary tumours and their secreted factors. Cascades targeting osteocytes can activate two pathways of malignancy: either osteoblastic (bone-forming) or osteolytic (bone-degrading) metastasis. Lysyl oxidase (LOX) secretion has been implicated in breast cancer, where under hypoxic conditions the ECM-modifying enzyme promotes osteolytic lesions^118–120^. Other ECM remodelling enzymes, such as MMPs derived from prostate cancer cells, are also involved in bone metastasis by activating osteoblast differentiation *via* NFκB signalling (Figure 3d)^121^. Strikingly, nutrient availability can determine the level of ECM remodelling that occurs at metastatic sites. Elia *et al*. found that breast cancer cells require the nutrient pyruvate to cause ECM remodelling *via* collagen hydroxylation at metastatic lung sites^122^. When pyruvate metabolism was inhibited in pre-clinical models of breast cancer, lung metastasis was significantly impaired^122^.

In melanoma, breast and lung cancer, the brain is a favoured organ of metastasis^123^; however, these cancers can also metastasise to other organs. Distinct from all other organs, the blood–brain barrier (BBB) offers an additional layer of protection from CTCs and immune cells; therefore, establishment of the PMN of the brain requires the breakdown of this barrier by secreted factors. Molecular mechanisms occurring at this boundary have previously had limited clarity due to lack of available human samples. Recently, Klotz and colleagues identified an interaction between luminal breast cancer-derived semaphorin (SEMA4D) and Plexin-B1 on human brain microvascular cells, which positively transmigrated CTCs through the BBB^124^. Interestingly, SEMA4D has also been involved in the formation of bone metastases, suggesting a common PMN regulator of this aberrantly secreted semaphorin transmembrane protein^125^. Overall, metastatic organotropism is yet another complex process often governed by cancer cell secretions, influencing both their immediate and their distant microenvironment. As highlighted, pathways initiating the choice of PMN are cancer type dependent and in some cases can override other cancer types to coerce an atypical secondary site^108^. This suggests a varying metastatic degree exists between cancer cell types, and the transfer of metastatic potential between cancer cells could be an interesting topic to consider in the future.

### Vasculature remodelling at the pre-metastatic niche

Another crucial factor of PMN formation is the remodelling of vasculature at the PMN organ *via* primary tumour-secreted factors. Altered vascularisation of the PMN means permeability is aberrantly increased, allowing enhanced penetrance of circulating pro-tumourigenic factors into the target organ (Figure 3c). This pathophysiological reorganisation has been associated with increased metastatic burden in several cancers^126,127^. VEGF is a well-characterised tumour-derived secretory factor involved in the endothelial reorganisation of the PMN through the activation of BMDCs and a multitude of signalling pathways^100,128^. More recent work has established that downstream targets of VEGF become compromised, which promotes tight junction disruption and hyperpermeability observed in PMN^129,130^. Occludin, a transmembrane protein that regulates tight junctions, was found to be downregulated in the pre-metastatic lung from a metastatic breast cancer murine model^129,130^. This was suggested to occur from cancer cell-derived VEGF-stimulated phosphorylation and ubiquitination of occludin, therefore impacting the tight junction-regulating function of occludin. Another recent study has suggested that vascular leakiness as well as angiogenesis in the PMN can be observed prior to the arrival of CTCs^131^. Although this is not currently considered a step in the “metastatic cascade”, the authors present the idea that metastasis can occur very rapidly without a transient stage of dormancy and therefore PMN angiogenesis should be considered as a crucial player in metastatic success^131^. Using an orthotopic model of metastatic breast cancer, the authors showed accumulation of MDSCs in the lungs during PMN formation but before cancer cell arrival^131^. This has been previously established^132,133^; however, this study shows that the recruitment of MDSCs by cancer cell-derived IL-1β and TNF secretion could aid in PMN angiogenesis by revealing a number of pro-angiogenic factors secreted by MDSCs, including MMP-9^131^. Furthermore, they showed this recruitment of MDSCs is regulated by a complement-dependent pathway, complement C5a receptor 1–MDSC (C5aR1–MDSC) axis, and, when pharmacologically blocked, pro-angiogenic factors were reduced, vascular density diminished, anti-tumour immunity improved, and ultimately metastatic burden was lessened^131^. This is especially exciting, as C5aR1 blockade can be combined with currently used immunotherapies such as *Listeria monocytogenes*-based vaccines, which aim to stimulate T cell responses to tumour and metastatic vasculature formation^134,135^. In combination, efficacy was improved and showed more success in prohibiting metastases than the standard-of-care drug sunitinib, a pan-inhibitor of VEGFR^131^. Studies such as this exemplify the benefit of stepping outside of the previously established PMN paradigm, offering hope for future alternative cancer therapies as a result.

### ECM remodelling at the pre-metastatic niche

As well as the altered vasculature, the ECM undergoes extensive remodelling at the PMN to house invading and expanding cancer cells. This occurs in two processes and is directed by the primary tumour: the depositing of new ECM components and the degradation of the pre-existing ECM. Activated fibroblasts and MDSCs play a large role in establishing the cancer-permissive landscape (Figure 3d). A recent study found that the stress-induced p38α protein kinase (encoded by *Mapk14)* is activated in lung fibroblasts by metastatic malignant cell secretory factors^136^. Activation of lung fibroblasts caused a cascade of pro-metastatic downstream effects, including repression of IFNAR1 (IFN α/β receptor subunit 1), which has previously been shown to be inhibitory towards PMN formation in metastatic melanoma models^137^. Fibroblast activation protein (FAP) expression was also induced in the activated fibroblasts, resulting in excessive fibronectin deposition and neutrophil recruitment *via* cancer cell-secreted CXCL1^136^. Preparation of the PMN ECM has implicated more than the cancer cell secretome, and recent evidence shows that CAFs within the primary tumour stroma are able to release factors that induce fibroblasts in the distant PMN of the lung^138^. CAF-derived EVs were detected by lung fibroblasts in the PMN, mediated by integrin α_2_β_1_^138^. This triggered TGF-β_2_ signalling pathways in lung fibroblasts and prompted extensive remodelling of lung ECM. EVs released by CAFs were shown to be more influential, in this case, in remodelling the PMN ECM over EVs released by cancer cells^138^. These studies offer a new perspective for ECM remodelling of the PMN, and shows that the cancer cell is capable of manipulating the systemic environment for tumour growth advantage on many levels. Perhaps this is indicative of a mesenchymal-common response reverberating through the systemic cancer-associated secretome, which is a powerful tool to harness for the future.

## Future perspectives

Taken together, these studies and others have shown that cancer cell-derived secretions are implicated in all steps of disease induction, development, and progression. However, many mechanisms through which cancer achieves these processes are still being elucidated. Through increased availability of pre-clinical and patient samples, recent research has highlighted just how complex and intertwined cancer pathways can become when interacting with the tumour stroma and the PMN. Although it was only briefly discussed in this review, many other factors in the TME can dictate the progression of disease. For example, the influence of heterogenous subpopulations of CAFs recently described by Ohlund *et al*.^73^ and others^74,139^ have been shown to have diverse roles in cancer promotion and therapy resistance. Moreover, biomechanical cues from collagen reorganisation in the desmoplastic stroma can largely impact proliferation and migration^140^. These stromal aberrations are first activated by the cancer cell, but the dynamic crosstalk between the neoplastic cell and the stromal environment has a monumental impact on cancer progression. These supportive cells are just as essential for the disease to proceed, and new discoveries between cancer and matrix are frequently being revealed.

Furthermore, the intratumoural heterogeneity of patient tumours poses another challenge in the treatment of cancer. Genetically engineered mouse models are essential for biomedical research, yet they often harbour specific global mutations that do not fully align with the genotype of patient tumours. New evidence is attempting to “map” a more realistic version by highlighting the inter-clonal communication that can occur between cancer cells, therefore highlighting distinct and potentially targetable secretions arising from heterogenous cancer cell populations^141,142^. Additionally, lineage plasticity may be an emerging pathway for therapy resistance that is shared between cancers. This survival mechanism allows the cancer cell to adapt to an altered metabolic state induced by therapy, such as hypoxia, thereby facilitating a change in histological phenotype and renders it unresponsive to the targeted therapy^143^. Recent research suggests that this does not necessarily mean a complete switch of phenotype to a canonical lineage. Instead the altered cancer cell has been shown to take on hybrid or new lineages, which could be driven by therapy-induced epigenetic changes^143–145^, highlighting the importance of cancer cell plasticity in treatment longevity.

Although outside the scope of this review, the therapy-induced secretome plays a significant role in therapy resistance. Often anti-cancer therapies aim to impede cancer progression by inducing senescence, a cellular state in which cells do not proliferate and become stalled in the cell cycle. Although this concept is initially advantageous in reducing tumour burden, it can often have the unintended side effect of inducing a senescence-associated secretory phenotype (SASP) in cancer cells and non-transformed cells of the TME^146^. SASP-affected cells paradoxically secrete high levels of pro-inflammatory cytokines, chemokines, proteases, growth factors, and EVs owing to persistent DNA damage response (DDR), which, in the context of malignancy, can be pro- or anti-tumourigenic^146–148^. Further work is required to elucidate the secretome-dependent mechanisms by which tumours avoid anti-cancer treatments. Overall, research and technological advances in the field of cancer cell secretomics are absolutely essential to expand our current understanding of cancer, from initiation to overcoming therapy resistance. By doing so, it will expand and connect the network of different cancer research areas, allowing for multidisciplinary and novel discoveries in the future of medical health.

## References

[1] Tjalsma H Bolhuis A Jongbloed JD: Signal peptide-dependent protein transport in Bacillus subtilis: A genome-based survey of the secretome. *Microbiol Mol Biol Rev.* 2000; 64(3): 515–47. 10.1128/mmbr.64.3.515-547.2000 10974125 PMC99003

[2] Zhang M Schekman R: Cell biology. Unconventional secretion, unconventional solutions. *Science.* 2013; 340(6132): 559–61. 10.1126/science.1234740 23641104

[3] Kim J Gee HY Lee MG: Unconventional protein secretion - new insights into the pathogenesis and therapeutic targets of human diseases. *J Cell Sci.* 2018; 131(12): jcs213686. 10.1242/jcs.213686 29941450

[4] Uhlén M Karlsson MJ Hober A: The human secretome. *Sci Signal.* 2019; 12(609): eaaz0274. 10.1126/scisignal.aaz0274 31772123

[5] Zhang H Freitas D Kim HS: Identification of distinct nanoparticles and subsets of extracellular vesicles by asymmetric flow field-flow fractionation. *Nat Cell Biol.* 2018; 20(3): 332–43. 10.1038/s41556-018-0040-4 29459780 PMC5931706

[6] Novo D Heath N Mitchell L: Mutant p53s generate pro-invasive niches by influencing exosome podocalyxin levels. *Nat Commun.* 2018; 9(1): 5069. 10.1038/s41467-018-07339-y 30498210 PMC6265295

[7] Verweij FJ Revenu C Arras G: Live Tracking of Inter-organ Communication by Endogenous Exosomes *In Vivo*. *Dev Cell.* 2019; 48(4): 573–589.e4. 10.1016/j.devcel.2019.01.004 30745143

[8] Follain G Herrmann D Harlepp S: Fluids and their mechanics in tumour transit: Shaping metastasis. *Nat Rev Cancer.* 2020; 20(2): 107–24. 10.1038/s41568-019-0221-x 31780785

[9] Brown KJ Seol H Pillai DK: The human secretome atlas initiative: Implications in health and disease conditions. *Biochim Biophys Acta.* 2013; 1834(11): 2454–61. 10.1016/j.bbapap.2013.04.007 23603790 PMC3755092

[10] Karagiannis GS Pavlou MP Diamandis EP: Cancer secretomics reveal pathophysiological pathways in cancer molecular oncology. *Mol Oncol.* 2010; 4(6): 496–510. 10.1016/j.molonc.2010.09.001 20934395 PMC5527923

[11] Brandi J Manfredi M Speziali G: Proteomic approaches to decipher cancer cell secretome. *Semin Cell Dev Biol.* 2018; 78: 93–101. 10.1016/j.semcdb.2017.06.030 28684183

[12] Simons M Raposo G: Exosomes--vesicular carriers for intercellular communication. *Curr Opin Cell Biol.* 2009; 21(4): 575–81. 10.1016/j.ceb.2009.03.007 19442504

[13] Uhlén M Fagerberg L Hallström BM: Proteomics. Tissue-based map of the human proteome. *Science.* 2015; 347(6220): 1260419. 10.1126/science.1260419 25613900

[14] Blanco MA LeRoy G Khan Z: Global secretome analysis identifies novel mediators of bone metastasis. *Cell Res.* 2012; 22(9): 1339–55. 10.1038/cr.2012.89 22688892 PMC3434351

[15] Mustafa S Pan L Marzoq A: Comparison of the tumor cell secretome and patient sera for an accurate serum-based diagnosis of pancreatic ductal adenocarcinoma. *Oncotarget.* 2017; 8(7): 11963–76. 10.18632/oncotarget.14449 28060763 PMC5355318

[16] Pappa KI Kontostathi G Makridakis M: High Resolution Proteomic Analysis of the Cervical Cancer Cell Lines Secretome Documents Deregulation of Multiple Proteases. *Cancer Genomics Proteomics.* 2017; 14(6): 507–21. 10.21873/cgp.20060 29109100 PMC6070321

[17] Robinson JL Feizi A Uhlén M: A Systematic Investigation of the Malignant Functions and Diagnostic Potential of the Cancer Secretome. *Cell Rep.* 2019; 26(10): 2622–2635.e5. 10.1016/j.celrep.2019.02.025 30840886 PMC6441842

[18] Bonnans C Chou J Werb Z: Remodelling the extracellular matrix in development and disease. *Nat Rev Mol Cell Biol.* 2014; 15(12): 786–801. 10.1038/nrm3904 25415508 PMC4316204

[19] Page-McCaw A Ewald AJ Werb Z: Matrix metalloproteinases and the regulation of tissue remodelling. *Nat Rev Mol Cell Biol.* 2007; 8(3): 221–33. 10.1038/nrm2125 17318226 PMC2760082

[20] Wang YZ Wu KP Wu AB: MMP-14 overexpression correlates with poor prognosis in non-small cell lung cancer. *Tumour Biol.* 2014; 35(10): 9815–21. 10.1007/s13277-014-2237-x 24986569

[21] Yang B Tang F Zhang B: Matrix metalloproteinase-9 overexpression is closely related to poor prognosis in patients with colon cancer. *World J Surg Oncol.* 2014; 12: 24. 10.1186/1477-7819-12-24 24476461 PMC3906768

[22] Ren F Tang R Zhang X: Overexpression of MMP Family Members Functions as Prognostic Biomarker for Breast Cancer Patients: A Systematic Review and Meta-Analysis. *PLoS One.* 2015; 10(8): e0135544. 10.1371/journal.pone.0135544 26270045 PMC4535920

[23] Lu P Takai K Weaver VM: Extracellular matrix degradation and remodeling in development and disease. *Cold Spring Harb Perspect Biol.* 2011; 3(12): a005058. 10.1101/cshperspect.a005058 21917992 PMC3225943

[24] Gialeli C Theocharis AD Karamanos NK: Roles of matrix metalloproteinases in cancer progression and their pharmacological targeting. *FEBS J.* 2011; 278(1): 16–27. 10.1111/j.1742-4658.2010.07919.x 21087457

[25] Gobin E Bagwell K Wagner J: A pan-cancer perspective of matrix metalloproteases (MMP) gene expression profile and their diagnostic/prognostic potential. *BMC Cancer.* 2019; 19(1): 581. 10.1186/s12885-019-5768-0 31200666 PMC6567474

[26] Sahin U Weskamp G Kelly K: Distinct roles for ADAM10 and ADAM17 in ectodomain shedding of six EGFR ligands. *J Cell Biol.* 2004; 164(5): 769–79. 10.1083/jcb.200307137 14993236 PMC2172154

[27] Levin M Udi Y Solomonov I: Next generation matrix metalloproteinase inhibitors - Novel strategies bring new prospects. *Biochim Biophys Acta Mol Cell Res.* 2017; 1864(11 Pt A): 1927–39. 10.1016/j.bbamcr.2017.06.009 28636874

[28] Fields GB: The Rebirth of Matrix Metalloproteinase Inhibitors: Moving Beyond the Dogma. *Cells.* 2019; 8(9): 984. 10.3390/cells8090984 31461880 PMC6769477

[29] Morton JP Karim SA Graham K: Dasatinib inhibits the development of metastases in a mouse model of pancreatic ductal adenocarcinoma. *Gastroenterology.* 2010; 139(1): 292–303. 10.1053/j.gastro.2010.03.034 20303350

[30] Egeblad M Werb Z: New functions for the matrix metalloproteinases in cancer progression. *Nat Rev Cancer.* 2002; 2(3): 161–74. 10.1038/nrc745 11990853

[31] Scilabra SD Pigoni M Pravatá V: Increased TIMP-3 expression alters the cellular secretome through dual inhibition of the metalloprotease ADAM10 and ligand-binding of the LRP-1 receptor. *Sci Rep.* 2018; 8(1): 14697. 10.1038/s41598-018-32910-4 30279425 PMC6168507

[32] Atapattu L Saha N Chheang C: An activated form of ADAM10 is tumor selective and regulates cancer stem-like cells and tumor growth. *J Exp Med.* 2016; 213(9): 1741–57. 10.1084/jem.20151095 27503072 PMC4995075

[33] Li Z Li AD Xu L: SPARC expression in gastric cancer predicts poor prognosis: Results from a clinical cohort, pooled analysis and GSEA assay. *Oncotarget.* 2016; 7(43): 70211–22. 10.18632/oncotarget.12191 28053291 PMC5342547

[34] John B Naczki C Patel C: Regulation of the bi-directional cross-talk between ovarian cancer cells and adipocytes by SPARC. *Oncogene.* 2019; 38(22): 4366–83. 10.1038/s41388-019-0728-3 30765860 PMC6542715

[35] Kessenbrock K Plaks V Werb Z: Matrix metalloproteinases: regulators of the tumor microenvironment. *Cell.* 2010; 141(1): 52–67. 10.1016/j.cell.2010.03.015 20371345 PMC2862057

[36] Patan S Munn LL Jain RK: Intussusceptive microvascular growth in a human colon adenocarcinoma xenograft: a novel mechanism of tumor angiogenesis. *Microvasc Res.* 1996; 51(2): 260–72. 10.1006/mvre.1996.0025 8778579

[37] Jain RK: Molecular regulation of vessel maturation. *Nat Med.* 2003; 9(6): 685–93. 10.1038/nm0603-685 12778167

[38] Krishna Priya S Nagare RP Sneha VS: Tumour angiogenesis-Origin of blood vessels. *Int J Cancer.* 2016; 139(4): 729–35. 10.1002/ijc.30067 26934471

[39] Bergers G Benjamin LE: Tumorigenesis and the angiogenic switch. *Nat Rev Cancer.* 2003; 3(6): 401–10. 10.1038/nrc1093 12778130

[40] Tonini T Rossi F Claudio PP: Molecular basis of angiogenesis and cancer. *Oncogene.* 2003; 22(42): 6549–56. 10.1038/sj.onc.1206816 14528279

[41] Nyberg P Salo T Kalluri R: Tumor microenvironment and angiogenesis. *Front Biosci.* 2008; 13: 6537–53. 10.2741/3173 18508679

[42] De Palma M Biziato D Petrova TV: Microenvironmental regulation of tumour angiogenesis. *Nat Rev Cancer.* 2017; 17(8): 457–74. 10.1038/nrc.2017.51 28706266

[43] Fukumura D Xu L Chen Y: Hypoxia and acidosis independently up-regulate vascular endothelial growth factor transcription in brain tumors *in vivo*. *Cancer Res.* 2001; 61(16): 6020–4. 11507045

[44] Shi Q Le X Wang B: Regulation of vascular endothelial growth factor expression by acidosis in human cancer cells. *Oncogene.* 2001; 20(28): 3751–6. 10.1038/sj.onc.1204500 11439338

[45] Corbet C Feron O: Tumour acidosis: from the passenger to the driver's seat. *Nat Rev Cancer.* 2017; 17(10): 577–93. 10.1038/nrc.2017.77 28912578

[46] Patton MC Zubair H Khan MA: Hypoxia alters the release and size distribution of extracellular vesicles in pancreatic cancer cells to support their adaptive survival. *J Cell Biochem.* 2020; 121(1): 828–39. 10.1002/jcb.29328 31407387 PMC6878126

[47] Hsu YL Hung JY Chang WA: Hypoxic lung cancer-secreted exosomal miR-23a increased angiogenesis and vascular permeability by targeting prolyl hydroxylase and tight junction protein ZO-1. *Oncogene.* 2017; 36(34): 4929–42. 10.1038/onc.2017.105 28436951

[48] Li J Yuan H Xu H: Hypoxic Cancer-Secreted Exosomal miR-182-5p Promotes Glioblastoma Angiogenesis by Targeting Kruppel-like Factor 2 and 4. *Mol Cancer Res.* 2020; 18(8): 1218–31. 10.1158/1541-7786.MCR-19-0725 32366676

[49] Cho JA Park H Lim EH: Exosomes from breast cancer cells can convert adipose tissue-derived mesenchymal stem cells into myofibroblast-like cells. *Int J Oncol.* 2012; 40(1): 130–8. 10.3892/ijo.2011.1193 21904773

[50] Follain G Osmani N Azevedo AS: Hemodynamic Forces Tune the Arrest, Adhesion, and Extravasation of Circulating Tumor Cells. *Dev Cell.* 2018; 45(1): 33–52.e12. 10.1016/j.devcel.2018.02.015 29634935

[51] Follain G Osmani N Mercier L: Impairing flow-mediated endothelial remodeling reduces extravasation of tumor cells. *bioRxiv.* 2020; 2020.07.27.219568 10.1101/2020.07.27.219568 PMC8222393 34162963

[52] Motz GT Coukos G: The parallel lives of angiogenesis and immunosuppression: Cancer and other tales. *Nat Rev Immunol.* 2011; 11(10): 702–11. 10.1038/nri3064 21941296

[53] Khan KA Kerbel RS: Improving immunotherapy outcomes with anti-angiogenic treatments and vice versa. *Nat Rev Clin Oncol.* 2018; 15(5): 310–24. 10.1038/nrclinonc.2018.9 29434333

[54] Kumar V Patel S Tcyganov E: The Nature of Myeloid-Derived Suppressor Cells in the Tumor Microenvironment. *Trends Immunol.* 2016; 37(3): 208–20. 10.1016/j.it.2016.01.004 26858199 PMC4775398

[55] Wherry EJ: T cell exhaustion. *Nat Immunol.* 2011; 12(6): 492–9. 10.1038/ni.2035 21739672

[56] Vitale I Manic G Coussens LM: Macrophages and Metabolism in the Tumor Microenvironment. *Cell Metab.* 2019; 30(1): 36–50. 10.1016/j.cmet.2019.06.001 31269428

[57] Mantovani A Allavena P: The interaction of anticancer therapies with tumor-associated macrophages. *J Exp Med.* 2015; 212(4): 435–45. 10.1084/jem.20150295 25753580 PMC4387285

[58] Cassetta L Fragkogianni S Sims AH: Human Tumor-Associated Macrophage and Monocyte Transcriptional Landscapes Reveal Cancer-Specific Reprogramming, Biomarkers, and Therapeutic Targets. *Cancer Cell.* 2019; 35(4): 588–602.e10. 10.1016/j.ccell.2019.02.009 30930117 PMC6472943

[59] Teijeira Á Garasa S Gato M: CXCR1 and CXCR2 Chemokine Receptor Agonists Produced by Tumors Induce Neutrophil Extracellular Traps that Interfere with Immune Cytotoxicity. *Immunity.* 2020; 52(5): 856–871.e8. 10.1016/j.immuni.2020.03.001 32289253

[60] Albrengues J Shields MA Ng D: Neutrophil extracellular traps produced during inflammation awaken dormant cancer cells in mice. *Science.* 2018; 361(6409): eaao4227. 10.1126/science.aao4227 30262472 PMC6777850

[61] Lee W Ko SY Mohamed MS: Neutrophils facilitate ovarian cancer premetastatic niche formation in the omentum. *J Exp Med.* 2019; 216(1): 176–94. 10.1084/jem.20181170 30567719 PMC6314534

[62] Torcellan T Stolp J Chtanova T: *In Vivo* Imaging Sheds Light on Immune Cell Migration and Function in Cancer. *Front Immunol.* 2017; 8: 309. 10.3389/fimmu.2017.00309 28382036 PMC5360706

[63] Yazdani HO Roy E Comerci AJ: Neutrophil Extracellular Traps Drive Mitochondrial Homeostasis in Tumors to Augment Growth. *Cancer Res.* 2019; 79(21): 5626–39. 10.1158/0008-5472.CAN-19-0800 31519688 PMC6825588

[64] Steele CW Karim SA Leach JDG: CXCR2 Inhibition Profoundly Suppresses Metastases and Augments Immunotherapy in Pancreatic Ductal Adenocarcinoma. *Cancer Cell.* 2016; 29(6): 832–45. 10.1016/j.ccell.2016.04.014 27265504 PMC4912354

[65] Srivastava MK Sinha P Clements VK: Myeloid-derived suppressor cells inhibit T-cell activation by depleting cystine and cysteine. *Cancer Res.* 2010; 70(1): 68–77. 10.1158/0008-5472.CAN-09-2587 20028852 PMC2805057

[66] Groth C Hu X Weber R: Immunosuppression mediated by myeloid-derived suppressor cells (MDSCs) during tumour progression. *Br J Cancer.* 2019; 120(1): 16–25. 10.1038/s41416-018-0333-1 30413826 PMC6325125

[67] Rong Y Yuan CH Qu Z: Doxorubicin resistant cancer cells activate myeloid-derived suppressor cells by releasing PGE2. *Sci Rep.* 2016; 6: 23824. 10.1038/srep23824 27032536 PMC4817121

[68] Ghiringhelli F Puig PE Roux S: Tumor cells convert immature myeloid dendritic cells into TGF-beta-secreting cells inducing CD4^+^CD25^+^ regulatory T cell proliferation. *J Exp Med.* 2005; 202(7): 919–29. 10.1084/jem.20050463 16186184 PMC2213166

[69] Lee CR Kwak Y Yang T: Myeloid-Derived Suppressor Cells Are Controlled by Regulatory T Cells via TGF-β during Murine Colitis. *Cell Rep.* 2016; 17(12): 3219–32. 10.1016/j.celrep.2016.11.062 28009291

[70] Li C Jiang P Wei S: Regulatory T cells in tumor microenvironment: New mechanisms, potential therapeutic strategies and future prospects. *Mol Cancer.* 2020; 19(1): 116. 10.1186/s12943-020-01234-1 32680511 PMC7367382

[71] Wang X Lang M Zhao T: Cancer-FOXP3 directly activated CCL5 to recruit FOXP3^+^Treg cells in pancreatic ductal adenocarcinoma. *Oncogene.* 2017; 36(21): 3048–58. 10.1038/onc.2016.458 27991933 PMC5454319

[72] Kalluri R: The biology and function of fibroblasts in cancer. *Nat Rev Cancer.* 2016; 16(9): 582–98. 10.1038/nrc.2016.73 27550820

[73] Öhlund D Handly-Santana A Biffi G: Distinct populations of inflammatory fibroblasts and myofibroblasts in pancreatic cancer. *J Exp Med.* 2017; 214(3): 579–96. 10.1084/jem.20162024 28232471 PMC5339682

[74] Biffi G Oni TE Spielman B: IL1-Induced JAK/STAT Signaling Is Antagonized by TGFβ to Shape CAF Heterogeneity in Pancreatic Ductal Adenocarcinoma. *Cancer Discov.* 2019; 9(2): 282–301. 10.1158/2159-8290.CD-18-0710 30366930 PMC6368881

[75] Sahai E Astsaturov I Cukierman E: A framework for advancing our understanding of cancer-associated fibroblasts. *Nat Rev Cancer.* 2020; 20(3): 174–86. 10.1038/s41568-019-0238-1 31980749 PMC7046529

[76] Wu SZ Roden DL Wang C: Stromal cell diversity associated with immune evasion in human triple-negative breast cancer. *EMBO J.* 2020; 39(19): e104063. 10.15252/embj.2019104063 32790115 PMC7527929

[77] Biffi G Tuveson DA: Diversity and biology of cancer-associated fibroblasts. *Physiol Rev.* 2020; 101(1): 147–176. 10.1152/physrev.00048.2019 32466724 PMC7864232

[78] Öhlund D Elyada E Tuveson D: Fibroblast heterogeneity in the cancer wound. *J Exp Med.* 2014; 211(8): 1503–23. 10.1084/jem.20140692 25071162 PMC4113948

[79] Rhim AD Oberstein PE Thomas DH: Stromal elements act to restrain, rather than support, pancreatic ductal adenocarcinoma. *Cancer Cell.* 2014; 25(6): 735–47. 10.1016/j.ccr.2014.04.021 24856585 PMC4096698

[80] Mizutani Y Kobayashi H Iida T: Meflin-Positive Cancer-Associated Fibroblasts Inhibit Pancreatic Carcinogenesis. *Cancer Res.* 2019; 79(20): 5367–81. 10.1158/0008-5472.CAN-19-0454 31439548

[81] Jiang H Torphy RJ Steiger K: Pancreatic ductal adenocarcinoma progression is restrained by stromal matrix. *J Clin Invest.* 2020; 130(9): 4704–9. 10.1172/JCI136760 32749238 PMC7456216

[82] Vennin C Mélénec P Rouet R: CAF hierarchy driven by pancreatic cancer cell p53-status creates a pro-metastatic and chemoresistant environment via perlecan. *Nat Commun.* 2019; 10(1): 3637. 10.1038/s41467-019-10968-6 31406163 PMC6691013

[83] Pereira BA Vennin C Papanicolaou M: CAF Subpopulations: A New Reservoir of Stromal Targets in Pancreatic Cancer. *Trends Cancer.* 2019; 5(11): 724–41. 10.1016/j.trecan.2019.09.010 31735290

[84] Fiaschi T Marini A Giannoni E: Reciprocal metabolic reprogramming through lactate shuttle coordinately influences tumor-stroma interplay. *Cancer Res.* 2012; 72(19): 5130–40. 10.1158/0008-5472.CAN-12-1949 22850421

[85] Costa A Kieffer Y Scholer-Dahirel A: Fibroblast Heterogeneity and Immunosuppressive Environment in Human Breast Cancer. *Cancer Cell.* 2018; 33(3): 463–479.e10. 10.1016/j.ccell.2018.01.011 29455927

[86] Monteran L Erez N: The Dark Side of Fibroblasts: Cancer-Associated Fibroblasts as Mediators of Immunosuppression in the Tumor Microenvironment. *Front Immunol.* 2019; 10: 1835. 10.3389/fimmu.2019.01835 31428105 PMC6688105

[87] Unterleuthner D Neuhold P Schwarz K: Cancer-associated fibroblast-derived WNT2 increases tumor angiogenesis in colon cancer. *Angiogenesis.* 2020; 23(2): 159–77. 10.1007/s10456-019-09688-8 31667643 PMC7160098

[88] Hynes RO: The extracellular matrix: Not just pretty fibrils. *Science.* 2009; 326(5957): 1216–9. 10.1126/science.1176009 19965464 PMC3536535

[89] Santi A Kugeratski FG Zanivan S: Cancer Associated Fibroblasts: The Architects of Stroma Remodeling. *Proteomics.* 2018; 18(5–6): e1700167. 10.1002/pmic.201700167 29280568 PMC5900985

[90] Bin Lim S Chua MLK Yeong JPS: Pan-cancer analysis connects tumor matrisome to immune response. *NPJ Precis Oncol.* 2019; 3: 15. 10.1038/s41698-019-0087-0 31123708 PMC6531473

[91] Lu P Weaver VM Werb Z: The extracellular matrix: A dynamic niche in cancer progression. *J Cell Biol.* 2012; 196(4): 395–406. 10.1083/jcb.201102147 22351925 PMC3283993

[92] Najafi M Farhood B Mortezaee K: Extracellular matrix (ECM) stiffness and degradation as cancer drivers. *J Cell Biochem.* 2019; 120(3): 2782–90. 10.1002/jcb.27681 30321449

[93] Pickup MW Mouw JK Weaver VM: The extracellular matrix modulates the hallmarks of cancer. *EMBO Rep.* 2014; 15(12): 1243–53. 10.15252/embr.201439246 25381661 PMC4264927

[94] Malik R Lelkes PI Cukierman E: Biomechanical and biochemical remodeling of stromal extracellular matrix in cancer. *Trends Biotechnol.* 2015; 33(4): 230–6. 10.1016/j.tibtech.2015.01.004 25708906 PMC4380578

[95] Alexander J Cukierman E: Cancer associated fibroblast: Mediators of tumorigenesis. *Matrix Biol.* 2020; 91–92: 19–34. 10.1016/j.matbio.2020.05.004 32450219 PMC7434664

[96] Reid SE Kay EJ Neilson LJ: Tumor matrix stiffness promotes metastatic cancer cell interaction with the endothelium. *EMBO J.* 2017; 36(16): 2373–89. 10.15252/embj.201694912 28694244 PMC5556271

[97] Oudin MJ Jonas O Kosciuk T: Tumor Cell-Driven Extracellular Matrix Remodeling Drives Haptotaxis during Metastatic Progression. *Cancer Discov.* 2016; 6(5): 516–31. 10.1158/2159-8290.CD-15-1183 26811325 PMC4854754

[98] Tian C Clauser KR Öhlund D: Proteomic analyses of ECM during pancreatic ductal adenocarcinoma progression reveal different contributions by tumor and stromal cells. *Proc Natl Acad Sci U S A.* 2019; 116(39): 19609–18. 10.1073/pnas.1908626116 31484774 PMC6765243

[99] Høye AM Erler JT: Structural ECM components in the premetastatic and metastatic niche. *Am J Physiol Cell Physiol.* 2016; 310(11): C955–67. 10.1152/ajpcell.00326.2015 27053524

[100] Kaplan RN Riba RD Zacharoulis S: VEGFR1-positive haematopoietic bone marrow progenitors initiate the pre-metastatic niche. *Nature.* 2005; 438(7069): 820–7. 10.1038/nature04186 16341007 PMC2945882

[101] Peinado H Zhang H Matei IR: Pre-metastatic niches: Organ-specific homes for metastases. *Nat Rev Cancer.* 2017; 17(5): 302–17. 10.1038/nrc.2017.6 28303905

[102] Zomer A Maynard C Verweij FJ: In Vivo imaging reveals extracellular vesicle-mediated phenocopying of metastatic behavior. *Cell.* 2015; 161(5): 1046–57. 10.1016/j.cell.2015.04.042 26000481 PMC4448148

[103] Steenbeek SC Pham TV de Ligt J: Cancer cells copy migratory behavior and exchange signaling networks via extracellular vesicles. *EMBO J.* 2018; 37(15): e98357. 10.15252/embj.201798357 29907695 PMC6068466

[104] Gangoda L Liem M Ang CS: Proteomic Profiling of Exosomes Secreted by Breast Cancer Cells with Varying Metastatic Potential. *Proteomics.* 2017; 17(23–24). 10.1002/pmic.201600370 29115712

[105] Kalra H Gangoda L Fonseka P: Extracellular vesicles containing oncogenic mutant β-catenin activate Wnt signalling pathway in the recipient cells. *J Extracell Vesicles.* 2019; 8(1): 1690217. 10.1080/20013078.2019.1690217 31819794 PMC6883417

[106] Celià-Terrassa T Kang Y: Metastatic niche functions and therapeutic opportunities. *Nat Cell Biol.* 2018; 20(8): 868–77. 10.1038/s41556-018-0145-9 30050120

[107] Sceneay J Smyth MJ Möller A: The pre-metastatic niche: Finding common ground. *Cancer Metastasis Rev.* 2013; 32(3–4): 449–64. 10.1007/s10555-013-9420-1 23636348

[108] Hoshino A Costa-Silva B Shen TL: Tumour exosome integrins determine organotropic metastasis. *Nature.* 2015; 527(7578): 329–35. 10.1038/nature15756 26524530 PMC4788391

[109] Costa-Silva B Aiello NM Ocean AJ: Pancreatic cancer exosomes initiate pre-metastatic niche formation in the liver. *Nat Cell Biol.* 2015; 17(6): 816–26. 10.1038/ncb3169 25985394 PMC5769922

[110] Sleeman JP: The lymph node pre-metastatic niche. *J Mol Med (Berl).* 2015; 93(11): 1173–84. 10.1007/s00109-015-1351-6 26489604

[111] Ubellacker JM Tasdogan A Ramesh V: Lymph protects metastasizing melanoma cells from ferroptosis. *Nature.* 2020; 585(7823): 113–8. 10.1038/s41586-020-2623-z 32814895 PMC7484468

[112] Strilic B Offermanns S: Intravascular Survival and Extravasation of Tumor Cells. *Cancer Cell.* 2017; 32(3): 282–93. 10.1016/j.ccell.2017.07.001 28898694

[113] Brown M Assen FP Leithner A: Lymph node blood vessels provide exit routes for metastatic tumor cell dissemination in mice. *Science.* 2018; 359(6382): 1408–11. 10.1126/science.aal3662 29567714

[114] Pereira ER Kedrin D Seano G: Lymph node metastases can invade local blood vessels, exit the node, and colonize distant organs in mice. *Science.* 2018; 359(6382): 1403–7. 10.1126/science.aal3622 29567713 PMC6002772

[115] Lee E Fertig EJ Jin K: Breast cancer cells condition lymphatic endothelial cells within pre-metastatic niches to promote metastasis. *Nat Commun.* 2014; 5: 4715. 10.1038/ncomms5715 25178650 PMC4351998

[116] Seubert B Grünwald B Kobuch J: Tissue inhibitor of metalloproteinases (TIMP)-1 creates a premetastatic niche in the liver through SDF-1/CXCR4-dependent neutrophil recruitment in mice. *Hepatology.* 2015; 61(1): 238–48. 10.1002/hep.27378 25131778 PMC4280301

[117] Jablonska J Lang S Sionov RV: The regulation of pre-metastatic niche formation by neutrophils. *Oncotarget.* 2017; 8(67): 112132–44. 10.18632/oncotarget.22792 29340117 PMC5762385

[118] Erler JT Bennewith KL Cox TR: Hypoxia-induced lysyl oxidase is a critical mediator of bone marrow cell recruitment to form the premetastatic niche. *Cancer Cell.* 2009; 15(1): 35–44. 10.1016/j.ccr.2008.11.012 19111879 PMC3050620

[119] Cox TR Gartland A Erler JT: Lysyl Oxidase, a Targetable Secreted Molecule Involved in Cancer Metastasis. *Cancer Res.* 2016; 76(2): 188–92. 10.1158/0008-5472.CAN-15-2306 26732355

[120] Chitty JL Setargew YFI Cox TR: Targeting the lysyl oxidases in tumour desmoplasia. *Biochem Soc Trans.* 2019; 47(6): 1661–78. 10.1042/BST20190098 31754702

[121] Lynch CC Hikosaka A Acuff HB: MMP-7 promotes prostate cancer-induced osteolysis via the solubilization of RANKL. *Cancer Cell.* 2005; 7(5): 485–96. 10.1016/j.ccr.2005.04.013 15894268

[122] Elia I Rossi M Stegen S: Breast cancer cells rely on environmental pyruvate to shape the metastatic niche. *Nature.* 2019; 568(7750): 117–21. 10.1038/s41586-019-0977-x 30814728 PMC6451642

[123] Gállego Pérez-Larraya J Hildebrand J: Brain metastases. *Handb Clin Neurol.* 2014; 121: 1143–57. 10.1016/B978-0-7020-4088-7.00077-8 24365409

[124] Klotz R Thomas A Teng T: Circulating Tumor Cells Exhibit Metastatic Tropism and Reveal Brain Metastasis Drivers. *Cancer Discov.* 2020; 10(1): 86–103. 10.1158/2159-8290.CD-19-0384 31601552 PMC6954305

[125] Yang YH Buhamrah A Schneider A: Semaphorin 4D Promotes Skeletal Metastasis in Breast Cancer. *PLoS One.* 2016; 11(2): e0150151. 10.1371/journal.pone.0150151 26910109 PMC4766104

[126] Huang Y Song N Ding Y: Pulmonary vascular destabilization in the premetastatic phase facilitates lung metastasis. *Cancer Res.* 2009; 69(19): 7529–37. 10.1158/0008-5472.CAN-08-4382 19773447

[127] Hiratsuka S Goel S Kamoun WS: Endothelial focal adhesion kinase mediates cancer cell homing to discrete regions of the lungs via E-selectin up-regulation. *Proc Natl Acad Sci U S A.* 2011; 108(9): 3725–30. 10.1073/pnas.1100446108 21321210 PMC3048115

[128] Kaplan RN Rafii S Lyden D: Preparing the "soil": The premetastatic niche. *Cancer Res.* 2006; 66(23): 11089–93. 10.1158/0008-5472.CAN-06-2407 17145848 PMC2952469

[129] Jiang M Qin C Han M: Primary breast cancer induces pulmonary vascular hyperpermeability and promotes metastasis via the VEGF-PKC pathway. *Mol Carcinog.* 2016; 55(6): 1087–95. 10.1002/mc.22352 26152457

[130] Li R Qi Y Jiang M: Primary tumor-secreted VEGF induces vascular hyperpermeability in premetastatic lung via the occludin phosphorylation/ubiquitination pathway. *Mol Carcinog.* 2019; 58(12): 2316–26. 10.1002/mc.23120 31553086

[131] Ghouse SM Vadrevu SK Manne S: Therapeutic Targeting of Vasculature in the Premetastatic and Metastatic Niches Reduces Lung Metastasis. *J Immunol.* 2020; 204(4): 990–1000. 10.4049/jimmunol.1901208 31900334 PMC7012400

[132] Yan HH Pickup M Pang Y: Gr-1+CD11b+ myeloid cells tip the balance of immune protection to tumor promotion in the premetastatic lung. *Cancer Res.* 2010; 70(15): 6139–49. 10.1158/0008-5472.CAN-10-0706 20631080 PMC4675145

[133] Gao D Joshi N Choi H: Myeloid progenitor cells in the premetastatic lung promote metastases by inducing mesenchymal to epithelial transition. *Cancer Res.* 2012; 72(6): 1384–94. 10.1158/0008-5472.CAN-11-2905 22282653 PMC8543151

[134] Seavey MM Maciag PC Al-Rawi N: An anti-vascular endothelial growth factor receptor 2/fetal liver kinase-1 *Listeria monocytogenes* anti-angiogenesis cancer vaccine for the treatment of primary and metastatic Her-2/neu^+^ breast tumors in a mouse model. *J Immunol.* 2009; 182(9): 5537–46. 10.4049/jimmunol.0803742 19380802 PMC2850569

[135] Wood LM Paterson Y: Attenuated *Listeria monocytogenes*: A powerful and versatile vector for the future of tumor immunotherapy. *Front Cell Infect Microbiol.* 2014; 4: 51. 10.3389/fcimb.2014.00051 24860789 PMC4026700

[136] Gui J Zahedi F Ortiz A: Activation of p38α stress-activated protein kinase drives the formation of the pre-metastatic niche in the lungs. *Nat Cancer.* 2020; 1: 603–19. 10.1038/s43018-020-0064-0 PMC8194112 34124690

[137] Huangfu WC Qian J Liu C: Inflammatory signaling compromises cell responses to interferon alpha. *Oncogene.* 2012; 31(2): 161–72. 10.1038/onc.2011.221 21666722 PMC3175348

[138] Kong J Tian H Zhang F: Extracellular vesicles of carcinoma-associated fibroblasts creates a pre-metastatic niche in the lung through activating fibroblasts. *Mol Cancer.* 2019; 18(1): 175. 10.1186/s12943-019-1101-4 31796058 PMC6892147

[139] Elyada E Bolisetty M Laise P: Cross-Species Single-Cell Analysis of Pancreatic Ductal Adenocarcinoma Reveals Antigen-Presenting Cancer-Associated Fibroblasts. *Cancer Discov.* 2019; 9(8): 1102–23. 10.1158/2159-8290.CD-19-0094 31197017 PMC6727976

[140] Cox TR Erler JT: Remodeling and homeostasis of the extracellular matrix: Implications for fibrotic diseases and cancer. *Dis Model Mech.* 2011; 4(2): 165–78. 10.1242/dmm.004077 21324931 PMC3046088

[141] Mannello F Ligi D: Resolving breast cancer heterogeneity by searching reliable protein cancer biomarkers in the breast fluid secretome. *BMC Cancer.* 2013; 13: 344. 10.1186/1471-2407-13-344 23849048 PMC3721990

[142] Guo M van Vliet M Zhao J: Identification of functionally distinct and interacting cancer cell subpopulations from glioblastoma with intratumoral genetic heterogeneity. *Neurooncol Adv.* 2020; 2(1): vdaa061. 10.1093/noajnl/vdaa061 32642713 PMC7309246

[143] Quintanal-Villalonga Á Chan JM Yu HA: Lineage plasticity in cancer: A shared pathway of therapeutic resistance. *Nat Rev Clin Oncol.* 2020; 17(6): 360–71. 10.1038/s41571-020-0340-z 32152485 PMC7397755

[144] Welti J Sharp A Yuan W: Targeting Bromodomain and Extra-Terminal (BET) Family Proteins in Castration-Resistant Prostate Cancer (CRPC). *Clin Cancer Res.* 2018; 24(13): 3149–62. 10.1158/1078-0432.CCR-17-3571 29555663

[145] Sehrawat A Gao L Wang Y: LSD1 activates a lethal prostate cancer gene network independently of its demethylase function. *Proc Natl Acad Sci U S A.* 2018; 115(18): E4179–E4188. 10.1073/pnas.1719168115 29581250 PMC5939079

[146] Coppé JP Patil CK Rodier F: Senescence-associated secretory phenotypes reveal cell-nonautonomous functions of oncogenic RAS and the p53 tumor suppressor. *PLoS Biol.* 2008; 6(12): 2853–68. 10.1371/journal.pbio.0060301 19053174 PMC2592359

[147] Rao SG Jackson JG: SASP: Tumor Suppressor or Promoter? Yes! *Trends Cancer.* 2016; 2(11): 676–87. 10.1016/j.trecan.2016.10.001 28741506

[148] Faget DV Ren Q Stewart SA: Unmasking senescence: Context-dependent effects of SASP in cancer. *Nat Rev Cancer.* 2019; 19(8): 439–53. 10.1038/s41568-019-0156-2 31235879

